# Relic of the Past or Clinical Compass? Re-Evaluating the Diagnostic Utility of Proton Magnetic Resonance Spectroscopy (^1^H-MRS) in Encephalitis and Infectious CNS Diseases

**DOI:** 10.3390/jcm15218543

**Published:** 2026-11-02

**Authors:** Maciej Naumowicz, Radosław Zawadzki, Joanna Zajkowska, Bożena Kubas

**Affiliations:** 1 Department of Radiology, Medical University of Białystok, M. Skłodowskiej-Curie 24A, 15-276 Białystok, Poland; 2 Department of Infectious Diseases and Neuroinfections, Medical University of Białystok, Zurawia 14, 15-540 Białystok, Poland

**Keywords:** proton magnetic resonance spectroscopy, CNS infections, encephalitis, differential diagnosis, neurometabolites

## Abstract

Neuroimaging of inflammatory and infectious CNS diseases using conventional MRI has limitations, such as the challenging differential diagnosis of visible lesions, as well as the absence of neuroimaging abnormalities despite severe symptoms. The aim of this review is to evaluate the diagnostic utility of proton magnetic resonance spectroscopy (^1^H-MRS) as a complementary method in inflammatory conditions and CNS infections of viral, bacterial, autoimmune, parasitic, or fungal etiology. We searched the PubMed, Web of Science, and Scopus databases, including studies and case reports evaluating ^1^H-MRS across age groups in the acute, subacute, and chronic phases of CNS disorders. The review demonstrates that MRS can serve as a valuable, non-invasive method capable of revealing metabolic abnormalities within normal-appearing brain tissue on conventional MRI. Furthermore, ^1^H-MRS detects specific metabolites-including cytosolic amino acids, acetate, and succinate in bacterial abscesses, trehalose in fungal lesions, and guanidinoacetate in tuberculomas-assisting in their differentiation from neoplastic lesions. Neurometabolite levels may correlate with clinical status, symptom severity, and cognitive deficits, while longitudinal observations enable monitoring of disease progression and treatment response. Although ^1^H-MRS is limited by spectral overlap, it represents a valuable complement to conventional MRI; however, further longitudinal studies on larger patient cohorts are required.

## 1. Introduction

Inflammatory and infectious diseases of the central nervous system (CNS), including viral, bacterial, autoimmune, parasitic, and fungal disorders, represent significant diagnostic and therapeutic challenges. These challenges result from the wide spectrum of possible pathogens and the often nonspecific clinical manifestations [1]. Although standard magnetic resonance imaging (MRI) is valuable for evaluating structural CNS abnormalities, it has considerable limitations in diagnosing certain encephalitides, such as anti-NMDAR (anti-N-methyl-D-aspartate receptor) encephalitis and tick-borne encephalitis. In many patients with these conditions, MRI findings may be unremarkable despite clear clinical symptoms [2,3,4].

Conventional MRI also has limitations for focal infectious CNS lesions, where differentiating infectious entities from neoplastic lesions can be difficult because of their nonspecific imaging appearance and resemblance to other intracranial pathologies, including tumors. In these cases, additional MRI techniques may provide complementary information to support differential diagnosis [5].

Proton magnetic resonance spectroscopy (^1^H-MRS) is a non-invasive modality that evaluates the metabolic profile of brain tissue. Key metabolites detectable by ^1^H-MRS include N-acetylaspartate (NAA), an indicator of neuronal integrity; choline (Cho), which reflects membrane turnover; creatine (Cr), associated with cellular energy metabolism; myo-inositol (mI), a marker of glial activity; glutamate (Glu) and glutamine (Gln), involved in glutamatergic metabolism; lactate (Lac), indicative of anaerobic metabolism; and lipids (Lip), which may signal membrane breakdown and necrosis [6]. By assessing these metabolites, ^1^H-MRS provides metabolic data that can indirectly evaluate neuroinflammatory and neurodegenerative mechanisms. Additionally, ^1^H-MRS can detect specific metabolic products of microorganisms, including amino acids, acetate, succinate, and trehalose. Identifying these compounds may help diagnose specific CNS infections and distinguish them from other focal intracranial lesions [7].

This review summarizes current evidence on the application and diagnostic utility of ^1^H-MRS in inflammatory and infectious CNS diseases, with particular focus on characteristic metabolic profiles and the potential role of this technique in differential diagnosis.

## 2. Materials and Methods

The PubMed, Web of Science, and Scopus databases were searched using the combination of (1) AND (2) of the following keywords: (1) ‘magnetic resonance spectroscopy’, ‘MR spectroscopy’, ‘proton MR spectroscopy’, ‘^1^H MRS’, ‘MRS’, MRSI, MR spectroscopic imaging, CSI; and (2) ‘encephalitis’, ‘neuroinfection’, ‘autoimmune encephalitis’, ‘limbic encephalitis’, ‘viral encephalitis’, ‘parasitic CNS infection’, ‘brain abscess’, ‘fungal CNS infection’, ‘postinfectious encephalopathies’, ‘HIV-associated encephalopathy’, or ‘PML’.

Our review includes studies published between 1996 and 2026 (until 1 September 2026). We have included relevant articles in order to assess the utility of proton magnetic resonance spectroscopy among patients with inflammatory and infectious central nervous system disorders as accurately as possible. The included papers were original research studies or clinical case reports that used ^1^H -MRS with patients of all ages (both pediatric and adult) in acute, post-acute, or chronic stages of infectious, autoimmune, or inflammatory central nervous system disorders. A total of 63 unique publications, comprising original research studies and clinical case reports, were selected for the review. The included articles were analyzed with regard to: (1) disease-specific metabolic profiles identified by MRS; (2) the potential advantages of MRS over other neuroimaging modalities in the investigated conditions; (3) the potential role of MRS as an adjunctive tool in the differential diagnosis of these conditions; (4) the relationship between MRS abnormalities and the severity of clinical symptoms; (5) the potential use of MRS for monitoring disease progression and treatment response; and (6) the prognostic value of MRS findings and (7) methodological considerations, including echo times (short vs. long TE), voxel placement, and acquisition technique (single-voxel vs. multi-voxel).

We included data on the following: (1) deviations from normal values in spectral profiles of brain tissue and pathogen-derived metabolites, including NAA, Cho, Cr, mI, Lac, Lip, Glu, Gln, total creatine (tCr), Glu/Gln complex (Glx), cytosolic amino acids (alanine, leucine, valine), succinate, acetate, guanidinoacetate, trehalose, and pyruvate; (2) stage-dependent variations in metabolic profiles across acute, subacute, and chronic stages of the disease, as well as the reversibility of observed metabolic abnormalities following therapy; (3) the capability of MRS to identify diffuse, global, or occult metabolic alterations in normal-appearing brain tissue beyond focal lesions visible on conventional MRI; (4) the diagnostic sensitivity and specificity of MRS relative to other neuroimaging modalities; (5) correlations of individual metabolic peaks and metabolite ratios with clinical severity scores, functional tests, and overall patient prognosis; and (6) the spatial concordance between the localization of MRS metabolic alterations and conventional MRI findings.

## 3. Diagnostic Utility of MRS

### 3.1. Viral Encephalitis

#### 3.1.1. Herpes Simplex Encephalitis (HSE)

Previous studies using MRS in the acute phase of HSE have shown reduced NAA levels and a decreased NAA/Cr ratio, indicating a loss of neuronal integrity associated with the acute inflammatory process in affected areas [8,9]. However, these values partially or fully returned to normal within one year after disease onset [9,10]. In addition, the acute phase of the disease was defined by increased Cho levels and an elevated Cho/Cr ratio, associated with macrophage infiltration, as well as increased mI levels and an elevated mI/Cr ratio, reflecting pronounced gliosis. During the acute phase of necrotizing encephalitis, elevated lactate and lipid levels have also been reported [8].

It is worth noting that the studies and clinical case reports included in the present review (as summarized in Table 1) differ in terms of technical acquisition parameters, which are often not reported at all. These discrepancies may have affected the comparability and interpretation of the findings across individual studies. In the context of HSE, the previously cited study by Sämann et al. [8] reported elevated lactate levels during the acute phase of HSE and increased mI levels during follow-up examinations, whereas Takanashi et al. did not describe such changes in their patient [9]. However, this does not necessarily indicate that these metabolic alterations were absent in the patient reported by Takanashi et al. The long echo time (TE) used in their study (136 ms) limited the reliable assessment of mI and lipids and introduced the risk of Lac peak inversion [11].

Moreover, MRS has shown that HSE is a generalized process not confined to the temporal lobe affected by the disease on conventional MRI. Hitosugi et al. also demonstrated metabolic abnormalities in the contralateral temporal lobe, including a mild decrease in the NAA/Cr ratio and an increase in the Cho/Cr ratio [12].

The pattern of metabolic abnormalities observed in HSE provides insight into the disease’s pathophysiology. It may help assess the severity and reversibility of neurodegeneration, the degree of gliosis, and the extent of necrosis. However, MRS has limited diagnostic value in differentiating HSE from other conditions, as the metabolic profile can resemble that of other encephalitides and gliomas, including decreased NAA and increased Cho, Lac, and lipid levels [13].

#### 3.1.2. Tick-Borne Encephalitis (TBE)

The study by Zawadzki et al. on TBE showed that although conventional MRI findings are often normal, MRS may disclose significant metabolic abnormalities in the central nervous system [14]. Patients in the acute phase of TBE exhibited a decreased NAA/Cr ratio in both thalami and in the right basal ganglia, as well as an increased Cho/Cr ratio in the left thalamus and the left cerebellar hemisphere compared with healthy controls.

The study was performed using a long echo time (TE = 135 ms), which facilitated the assessment of signals from major metabolites, including NAA, Cho, and Cr, but limited the detailed characterization of the overall metabolic profile, particularly with regard to myo-inositol (mI) and glutamate–glutamine (Glx). This represents an area that warrants further investigation. In addition, studies using multi-voxel proton MR spectroscopy, or magnetic resonance spectroscopic imaging (MRSI), could provide valuable complementary information, as Zawadzki et al. used single-voxel spectroscopy (SVS), with voxels positioned only in selected brain structures. This is particularly relevant given that conventional MRI revealed no structural abnormalities in the patients studied.

Considering that nearly half of patients with TBE develop post-encephalitic syndrome (PES), characterized by persistent symptoms such as cognitive impairment and neurological deficits that may last for many years after the acute phase of TBE, longitudinal studies comparing metabolic profiles between patients who develop PES and those who do not appear particularly warranted. Another important research direction would be to investigate the associations between metabolic profiles and clinical symptom severity.

### 3.2. Autoimmune and Paraneoplastic Encephalitis

#### 3.2.1. Anti-NMDAR Encephalitis

Anti-NMDAR encephalitis is often associated with a serious clinical course, although conventional MRI does not show any abnormalities in about half of the cases; this is referred to as the clinical-radiological paradox [69].

Splendiani et al. reported the case of a 17-year-old patient who had been diagnosed with anti-NMDAR encephalitis and in whom the cerebellar lesions displayed a moderate decrease in the NAA peak (together with a moderate reduction in the NAA/Cr ratio), which suggests metabolic dysfunction or neuronal loss, and a moderate rise in the choline peak (along with a moderate increase in the Cho/Cr ratio), indicating an inflammatory process and increased cellularity [15]. The spectrum did not show any rise in lactate or lipid concentrations-findings that would point to necrosis or severe hypoxia. The authors stated that MRS can be of help in distinguishing an inflammatory process from high-grade neoplastic lesions (these being characterized by a considerably higher Cho/NAA ratio) and from HSE, in which a markedly reduced NAA concentration, greatly elevated choline peaks, and frequently detectable lactate and lipid peaks due to tissue necrosis are more commonly seen. Following treatment with methylprednisolone, the symptoms disappeared, and a follow-up spectroscopy carried out six months later revealed a complete normalization of the spectrum, showing that the initial changes were due to reversible neuronal damage and not to permanent necrosis. Kataoka et al. also described similar findings concerning a lowering of NAA levels during the acute phase of the disease in the basal ganglia and thalami, followed by a normalization of the metabolic profile in the later phase [16].

The observations described above concerned individual clinical cases; however, Hudasch et al. recently published a study describing the use of whole-brain MRS in 20 patients with anti-NMDAR encephalitis and 20 healthy controls [17]. The study showed that metabolic changes in anti-NMDAR encephalitis can be diffuse. A decreased NAA peak was observed in the right temporal lobe, an elevated Cho peak in the left frontal and parietal lobes as well as both occipital lobes, an elevated tCr peak in the left frontal and occipital lobes, and an increase in mI was noted in the left parietal lobe and both occipital lobes. Furthermore, MRS may help assess the patient’s clinical status, as Cho concentration in the right occipital lobe correlated with the results of the HPT for the left hand (fine motor skills test) and the walking speed test. Meanwhile, mI concentration in the right occipital lobe correlated with the disease severity evaluated by the NEOS score (anti-NMDAR encephalitis one-year functional status score). However, it is worth emphasizing that these results should be considered hypothesis-generating. For multi-voxel analysis, the 2019 ISMRM expert consensus (Wilson et al.), which defines current standards in ^1^H -MRS, requires rigorous Type I error control (e.g., FDR or Bonferroni correction) due to the large number of voxels compared [11]. In contrast, the study by Hudasch et al. utilized uncorrected Student’s *t*-tests, meaning these findings should be considered exploratory and require confirmation in larger, independently validated cohorts.

Fortea et al. examined 32 patients during the post-acute stage of anti-NMDAR encephalitis [18]. Their study found decreased levels of glutamate and glutamate + glutamine, along with elevated mI in the left dorsomedial prefrontal cortex (dmPFC) compared with healthy controls. No statistically significant differences in NAA concentrations were noted between the study group and the control group. Interestingly, the observed metabolic disturbances persisted throughout the entire follow-up period (24 months). The authors suggest that the persistent impairment of glutamatergic neurotransmission may result from an earlier increase in glutamate concentration during the acute phase of NMDARE caused by excessive activation of AMPA (α-amino-3-hydroxy-5-methyl-4-isoxazolepropionic acid) receptors by anti-NMDAR antibodies, which subsequently leads to their desensitization and lower glutamate concentration in the post-acute phase. Conversely, the increase in myo-inositol concentration is linked to astrocytic activation and persistent inflammation. Interestingly, this study did not show significant differences in the metabolic profile of the medial temporal lobe, a region commonly implicated in NMDARE [70]. However, it cannot be overlooked that the loss of a portion of the data due to low spectroscopic spectrum quality may have contributed to the lack of statistical significance [18]. The quality of the data could have been substantially affected by the use of the PRESS sequence, which is highly sensitive to magnetic field inhomogeneities (in this case related to the proximity of the paranasal sinuses), and the aforementioned expert consensus recommends using the semi-LASER/LASER sequence, explicitly noting that the application of PRESS at 3T can cause a significant chemical shift displacement error (CSDE).

To sum up, the present scientific evidence shows that MRS is able to detect metabolic changes which are not visible on standard MRI, thereby facilitating the resolution of the clinical-radiological paradox. Moreover, MRS can assist in distinguishing anti-NMDA receptor encephalitis from neoplastic processes, as well as in assessing the reversibility of neuronal damage, the patient’s clinical condition, and prognosis.

#### 3.2.2. Limbic Encephalitis

Limbic encephalitis (LE) is a clinical syndrome caused by an autoimmune or paraneoplastic process, characterized by a subacute course of symptoms involving the limbic system, along with structural and functional signs of damage to the medial temporal lobes [71]. Depending on the etiology, LE may be associated with antibodies against LGI1 (Leucine-rich glioma-inactivated 1), GABA-BR (Gamma-aminobutyric acid type B receptor), AMPAR, and CASPR2 (Contactin-associated protein-like 2), as well as intracellular antigens such as Hu and Ma2 [72].

##### Anti-LGI1 Encephalitis

The most common subtype of LE investigated in studies using MRS to date is anti-LGI1 encephalitis. Hang et al. reported that, in a patient with anti-LGI1 limbic encephalitis, they observed a moderate reduction in NAA and the NAA/Cr ratio, accompanied by a slight increase in Cho and the Cho/Cr ratio within the left hippocampus [19].

Larger study has focused on patients in the post-acute phase following LE. Szots et al. used MRS to examine nine patients who had experienced LGI1 antibody-associated encephalitis an average of 33 months earlier [20]. This form of encephalitis typically involves the limbic system, which was also demonstrated in the study population, with 8 of 9 patients developing hippocampal atrophy and sclerosis. However, when searching for metabolic abnormalities suggestive of more widespread brain involvement, the authors placed voxels in the right centrum semiovale and mid-occipital gray matter. The study found reduced glutamine/glutamate concentrations in the centrum semiovale white matter compared with healthy controls, indicating persistent alterations in the brain’s metabolic profile beyond the hippocampi. The study also noted lower NAA levels in the study group compared to the control group, although the difference was not statistically significant. The authors note that the mean age in both groups was approximately 60 years, suggesting that the lack of an observed difference could potentially be influenced by confounding aging effects.

##### Limbic Encephalitis Associated with Different Antibody Profiles

In patients who were in the post-acute phase of autoimmune encephalitis, Taraschenko and colleagues found that the concentration of myo-inositol was increased [21]. They observed alterations in the metabolic profile of the hippocampus (in AE patients with various antibodies (anti-NMDAR, anti-GAD65, anti-LGI1, and the seronegative form of AE)), but no such changes were seen in the prefrontal cortex. Methodologically, the study adheres to high-standard expert consensus guidelines for MRS by employing a short-echo semiLASER sequence (TE = 30 ms) and FASTMAP shimming, which ensured optimal spectral quality and minimized chemical shift displacement errors in the hippocampus.

Interestingly, a correlation was noted between a higher level of myo-inositol and poorer performance on memory recognition tests, as well as better attention in the patients. Furthermore, a correlation was found between a higher concentration of tCr in the hippocampus and a faster rate of information processing. This indicates that MRS might be able to reflect the functional consequences of previous encephalitis, and in the future myo-inositol could act as a potential biomarker for monitoring the recovery process and as a target for cognitive-protective therapies [21].

##### Paraneoplastic LE

To the best of our knowledge, MRS has been used in only one case of paraneoplastic limbic encephalitis. Isik et al. described a patient with anti-Hu antibodies in whom, during the early stage of the disease (3 weeks from symptom onset), they noted a slight decrease in NAA and an increase in mI in the right superior temporal neocortex. In the later stage, however, the authors demonstrated progressive neuronal loss (decreased NAA), increased gliosis (increased mI/Cr), accelerated membrane turnover (increased Cho/Cr), and anaerobic glycolysis (lactate peak) in the medial temporal lobe regions [22]. It is worth pointing out that this case report complied with the technical standards of the time (the paper was published in 2004). However, from the perspective of the current 2019 consensus, it exhibits several methodological limitations, namely: the use of the PRESS sequence instead of semi-LASER, the absence of an unsuppressed water reference scan, and the use of peak-based measurements rather than automated spectral fitting with a simulated metabolite basis set. Given these methodological limitations and the paucity of published evidence, further studies are needed to characterize MRS findings in paraneoplastic limbic encephalitis.

Villanueva-Castro et al., although they studied only patients with Low-Grade Glioma, emphasize the utility of MRS in differentiating LE from Low-Grade Glioma, as all patients who were ultimately diagnosed with a tumor presented to the hospital with suspected LE [23]. However, high Cho/NAA ratios, which averaged 2.3, suggested a neoplastic diagnosis. Importantly, this study did not specify the acquisition parameters due to a lack of standardization in the study protocols.

#### 3.2.3. Rasmussen’s Encephalitis

Rasmussen’s encephalitis is a chronic, progressive, and most often unilateral encephalitis, the pathomechanism of which has not been fully elucidated. Current data indicate that a T-cell-dependent immune response and microglial activation play an important role in the disease’s pathomechanism [73]. The disease leads to progressive atrophy of one cerebral hemisphere, permanent neurological deficits, and often drug-resistant epilepsy [73].

To date, research does not show evidence of abnormalities specific exclusively to this disease entity. As in the case of other types of encephalitis, MRS examination reveals a reduced NAA level and an elevated or normal Cho level, which leads to a decrease in the NAA/Cr and NAA/Cho ratios, suggesting neuronal loss or dysfunction [24,25]. Increase mI levels have also been reported [26,27].

In patients who have recently experienced uncontrolled epileptic seizures, a transient lactate peak has also been described [25,28,74]. It is also worth noting here that the metabolic profile in Rasmussen’s encephalitis fluctuates, and after status epilepticus, a transient decrease in the concentration of NAA, Cr, and Cho may also occur, as well as changes in Glx concentration [25].

Sundgren et al., utilizing multivoxel MRS, demonstrated that this technique may be more sensitive than conventional MRI for detecting brain tissue abnormalities in Rasmussen’s encephalitis [29]. In addition to abnormal metabolic profiles in the affected hemisphere, they observed decreased NAA/Cho and NAA/Cr ratios in the morphologically normal frontal periventricular white matter of the contralateral hemisphere. These abnormalities preceded the onset of neurological symptoms involving that hemisphere by 4 months.

Faria et al. also observed that metabolic changes in MRS may correlate with disease severity, as among patients with a milder disease course whose seizures were pharmacologically controlled, MRS showed no significant metabolic differences between the affected and contralateral cerebral hemispheres, whereas in cases with a more severe course, metabolic disturbances were observed, i.e., a decrease in NAA and an increase in Cho and Lac [28].

For the interpretation of the findings discussed above, it is important to acknowledge that the literature on the application of MRS in Rasmussen’s encephalitis is based predominantly on small, heterogeneous case series and individual case reports, which frequently lack standardized acquisition protocols and established quality control measures for spectral data.

#### 3.2.4. Summary

In summary, the available data indicate that MRS can show both focal and diffuse metabolic disturbances in autoimmune and paraneoplastic encephalitis. Depending on the disease entity and stage, the observed changes primarily include decreased NAA peaks, increased mI, and altered choline and Glu/Gln metabolism. Changes in brain tissue metabolic profiles during the post-acute phase of the disease, as well as their associations with the severity of cognitive deficits and clinical symptoms, appear particularly interesting. Moreover, reports suggest that MRS can detect metabolic abnormalities before the onset of overt clinical symptoms or the appearance of abnormalities on conventional MRI. In some cases, spectroscopic abnormalities normalized alongside clinical improvement, implying the possibility of reversibility of some observed disturbances. The lack of a specific MRS profile for individual entities, combined with a small number of studies and small sample sizes, limits the utility of MRS as a standalone diagnostic tool; however, this method can serve as an important adjunct to conventional MRI, supporting differential diagnosis.

### 3.3. Focal CNS Infections

#### 3.3.1. Bacterial Infections (Brain Abscess)

A brain abscess is an encapsulated collection of pus resulting as a complication of a focal intracerebral infection, most commonly caused by bacteria of the Staphylococcus and Streptococcus species [75].

In the case of bacterial brain abscesses, MRS can provide valuable clues in differentiating them from other cystic lesions, as it detects bacterial metabolic products and tissue degeneration products within the abscess [30,76]. Normally occurring tissue metabolites such as NAA, Cho, and Cr are significantly reduced or completely absent. Importantly, the presence of NAA, Cho, and Cr does not reflect the intrinsic metabolic profile of the abscess cavity itself, but rather stems from the partial volume effect.

In a spectrum obtained from a bacterial abscess prior to or in the initial phase of antibacterial therapy, characteristic amino acid peaks (alanine at 1.47 ppm, leucine at 0.95–1.05 ppm, and valine at 0.97–1.03 ppm) can be observed, which are products of protein proteolysis in the necrotic center of the abscess, alongside succinate peaks (2.4 ppm) and acetate peaks (1.9 ppm)-products of bacterial metabolism-and lactate (1.3 ppm), a product of anaerobic glycolysis [31,32]. Studies indicate that peaks corresponding to bacteria-associated metabolites (acetate, succinate, and pyruvate) disappear most rapidly during effective antimicrobial treatment [30,31]. Dev et al. additionally observed the disappearance of amino acid peaks following prolonged antibiotic therapy (4 weeks), with only the lactate peak remaining [31]. The peak at approximately 2.4 ppm is commonly attributed to succinate in the context of bacterial brain abscesses. However, it is worth noting that pyruvate signals may also occur in this spectral region. Therefore, metabolite identification based solely on chemical shift should be interpreted cautiously, particularly when spectral resolution and the signal-to-noise ratio (SNR) are limited [77]. A study of 194 patients showed that the amino acid peak was present in 80% of patients with an abscess, while the sensitivity and specificity of amino acids as an abscess marker in this study were reported as 0.72 and 0.30, respectively [34].

The presence of an amino acid peak can therefore be a sensitive marker for a bacterial abscess; however, the absence of amino acids cannot rule out a bacterial etiology. Studies indicate that the presence of an acetate peak, with or without an accompanying succinate peak, suggests that the infection is associated with an anaerobic bacterium [33], although these peaks can also be observed in certain facultative anaerobes [7,34]. Therefore, while MRS may facilitate the selection of more targeted antibiotic therapy, caution should be exercised when assigning etiologic agents to specific groups solely based on the spectroscopic spectrum. It is also worth noting that a brain abscess caused by S. aureus does not display the characteristic acetate, succinate, and amino acid peaks, and the spectral profile is dominated by lactate and lipid peaks, which, in the absence of an NAA peak, may mistakenly suggest a diagnosis of glioblastoma [35].

Additionally, it is useful to know that at 1.3 ppm, both lactate and lipid signals can overlap, making these metabolites difficult to distinguish; however, using two different echo times (TE 135 and 270 ms) solves this issue. In a spectrum acquired at TE 270 ms, amino acids appear as a positive peak at 1.3 ppm, whereas at TE 135 ms, this peak becomes inverted (negative), allowing it to be differentiated from the lipid peak, which remains positive at TE 135 ms [32].

To summarize the above findings and aid in the differential diagnosis of focal brain lesions, Table 2 has been provided. This table also highlights the difficulty in differentiating bacterial abscesses from neurocysticercosis, as these lesions may exhibit very similar metabolic profiles on MRS. However, a study by Agarwal et al. suggested that the presence and relative predominance of succinate and acetate peaks may be useful in differentiating these lesions [78]. Lesions associated with neurocysticercosis are characterized by the presence of succinate alone or by a higher succinate concentration than acetate. In contrast, anaerobic abscesses demonstrate either acetate alone or a higher acetate concentration than succinate.

#### 3.3.2. Mycobacterial Infections (Tuberculoma and Tuberculous Abscess)

MRS could be of use in distinguishing between a tuberculoma or a tuberculous abscess and other focal brain lesions since the spectrum shows a particular lipid resonance peak at 1.3 ppm. This occurs because of the high lipid content in the cell wall of Mycobacterium tuberculosis, as well as the abundance of fatty acids in the caseating necrotic material of the lesions [38,79,80,87].

Moreover, studies show that a guanidinoacetate peak at 3.8 ppm serves as a specific marker for tuberculomas and can therefore act as a potential biomarker for distinguishing tuberculomas from high-grade malignancies [39]. Tuberculomas that are highly cellular may show Cho peaks at 3.2 ppm [79]; however, as indicated by Parry et al., who utilized a multivoxel 3D CSI technique, the Cho/Cr and Cho/NAA ratios remain lower in tuberculomas than in high-grade gliomas and metastases [37]. Due to the simplified spectral analysis used in this study, which involved calculating metabolite ratios from the areas under the peaks rather than fitting the entire spectrum using parametric methods, and the lack of CSD correction, the measured Cho/Cr ratios may have been subject to measurement error. However, given the substantial differences in Cho/Cr ratios between tuberculomas (1.36), metastases (2.63), and gliomas (3.34), the overall diagnostic conclusion remains consistent with established neuroradiological knowledge. Nevertheless, further studies adhering to the latest ISMRM consensus recommendations are required to establish specific numerical cutoff values [11]. Furthermore, it is worth emphasizing that comparing metabolite ratios across different scientific reports remains challenging due to significant methodological discrepancies and the varying evolutionary stages of tuberculomas. Voxel placement (depending on whether it is positioned within the highly cellular, enhancing rim or the caseating core) and the choice of TE directly influence the relative levels of Cho, Cr, and NAA. Additionally, differences in analytical techniques account for further numerical discrepancies between studies, underscoring the need for standardized MRS acquisition protocols.

Tuberculous abscesses may exhibit a lactate peak [36,81]; nevertheless, in contrast to pyogenic bacterial abscesses, tuberculous abscesses do not have cytosolic amino acid resonances, nor do they show peaks of acetate and succinate [81]. Moreover, the lack of a trehalose peak (3.6–3.8 ppm) enables tuberculous abscesses to be distinguished from fungal abscesses [36].

It is difficult to tell the difference between a tuberculoma and a tuberculous abscess simply on the basis of MRS findings; yet it is stated that this distinction does not affect clinical management since in both cases antitubercular therapy is given and surgical treatment is carried out when needed [43].

Moreover, MRS has potential use in monitoring the response to antitubercular treatment since a progressive decrease in the lipid peak is observed after treatment has started. It acts as a valuable adjunct to conventional MRI when carrying out the diagnostic evaluation of tuberculomas and tuberculous abscesses, particularly in children, since the diagnosis is still difficult in this group [38].

#### 3.3.3. Parasitic Infections

##### Cerebral Toxoplasmosis

Elicer points out that MRS can be useful in differentiating focal lesions visible on conventional brain MRI in patients with HIV(Human Immunodeficiency Virus)/AIDS (Acquired Immunodeficiency Syndrome) [88]. They observed that lesions in toxoplasma encephalitis are characterized by markedly elevated Lip/Lac peaks around 1.3 ppm, accompanied by reduced peaks of other neurometabolites (NAA, creatine, choline). However, it is worth noting here that Lip/Lac peaks are not a specific marker for toxoplasmosis, as they result from necrosis and hypoxia, which can also occur in other disease entities (e.g., glioblastoma multiforme) [82].

Barcelo et al. also reported similar observations regarding the metabolic profile of cerebral toxoplasmosis when analyzing a single case of atypical cerebral toxoplasmosis with a single ring-enhancing lesion in the pons, where MRS helped differentiate a toxoplasma abscess from lesions associated with primary central nervous system lymphoma (PCNSL) [44]. The authors suggest that despite a similar profile of changes observed on MRS in these disease entities (most commonly associated with HIV/AIDS), a decrease or normal level of choline in cerebral toxoplasmosis may be a feature that distinguishes it from PCNSL, in which choline levels increase. However, Simone et al. point to the low specificity of MRS in differentiating toxoplasmosis from lymphoma, while emphasizing its high sensitivity in detecting metabolic brain tissue damage itself and the value of MRS in monitoring response to treatment [45]. A modern systematic review also confirmed that MRS has a limited ability to differentiate toxoplasmosis from PCNSL [82].

It is also worth emphasizing that choline levels in cerebral toxoplasmosis can be slightly elevated as well, which further complicates the differentiation from neoplastic lesions. Ferraz-Filho et al., after analyzing 42 cases of toxoplasmosis and 39 gliomas, suggested that concurrently considering Cho/Cr ratios > 1.97 and NAA/Cr < 1.12 allows for the detection of neoplasms with a specificity of 97.6% (using a TE of 144 ms) [46].

##### Neurocysticercosis

Batta et al. conducted a study to evaluate the utility of new MRI imaging techniques in diagnosing neurocysticercosis-a parasitic CNS infection caused by Taenia solium larvae [47]. The study included 85 patients, in whom the most common finding on conventional MRI was cystic, ring-enhancing lesions accompanied by mild edema. The authors state that MRI spectroscopy alone cannot serve as a standalone diagnostic method for neurocysticercosis because the lesions’ spectral profile is insufficiently characteristic. However, they point out the value of MRS in differentiating parasitic/inflammatory lesions from malignant neoplasms. They base these conclusions on observations in 24 patients, showing low Cho/Cr and Cho/NAA ratios (<2) inside 96% of the lesions, which argues against malignant neoplastic tumors [47]. However, it should be noted that the authors did not provide detailed technical parameters of the MRS acquisition, which limits the ability to assess the comparability of their results with those of other studies. Consequently, a value of 2 should not be considered a universal cutoff point for differentiating neurocysticercosis lesions from malignant neoplasms.

Additionally, unlike some neurocysticercosis studies [48,50,83], Batta et al. did not observe parasite-specific metabolites inside the lesions (such as alanine, succinate, or pyruvate) [47]; however, this may have been related to the small lesion size, which made precise voxel localization in MRS difficult. The described pyruvate peaks pertained to the racemose form of cysticercosis (racemose neurocysticercosis) and further help differentiate this form of the disease from cystic gliomas. Elevated lactate peaks within the lesions were also reported in some patients.

Hernández et al. also indicate that MRS can help distinguish neurocysticercosis lesions from tuberculomas [83]. The authors suggest that tuberculomas are characterized by significantly higher lipid peaks, choline peaks, and higher Cho/Cr ratios, as well as lower NAA peaks compared to neurocysticercosis [40,49,83]. Pretell et al. reported a Cho/Cr ratio greater than 1 in all tuberculomas (4/4), whereas none of the six neurocysticercosis lesions exceeded this threshold [40]. However, this value should be regarded as a preliminary discriminatory threshold rather than a validated diagnostic cutoff, given the small sample size and the lack of spectral fitting using simulated metabolite basis sets, as well as the absence of reported fitting quality metrics and measurement uncertainty. However, it is worth noting that elevated choline peaks apply to only a subset of tuberculomas (due to the inflammatory response in the lesion wall) [39]. In contrast, in most cases, the choline peak is low or absent [41].

Jayasundar et al. also compared neurocysticercosis lesions with tuberculomas [42]. The authors found very low levels of all metabolites and a low signal-to-noise ratio in NCC lesions. Additionally, they observed a statistically significant difference in the NAA/Cr ratio between the groups. This ratio appears to be exceptionally low compared to the study by Pretell et al. [40] (Table 2), but this fact may be explained by the different evolutionary stages of the studied lesions. Jayasundar et al. included patients with NCC whose lesions consisted of active cysts (vesicular stage) or calcifications. In contrast, Pretell et al. studied exclusively patients with single enhancing lesions containing degenerating parasites surrounded by an active inflammatory response.

#### 3.3.4. Fungal Infections

##### Cryptococcoma

CNS cryptococcosis is a fungal central nervous system infection caused by yeast of the Cryptococcus genus, primarily C. neoformans and C. gattii, occurring most commonly in immunocompromised individuals (HIV infection, post-transplantation, immunosuppressive therapy) [89,90]. A cryptococcoma, on the other hand, is a form of CNS cryptococcosis-more frequently observed in immunocompetent patients-in which the host’s granulomatous response forms a mass-like lesion within the brain parenchyma. In neuroimaging, this can mimic a primary brain tumor or metastasis [91].

Previously reported proton MRS findings in patients with cryptococcoma were non-specific, denoting an inflammatory process with neuronal loss and gliosis [51]. A decrease in NAA and Cr, as well as elevated lipid-lactate and Cho peaks, have been observed [51,84]. In some cases, very high Cho/NAA ratios were described, which can raise suspicion of a high-grade malignancy and complicate differential diagnosis [51,52]. However, crucially for differential diagnosis, numerous peaks between 3.6 and 3.8 ppm have been reported in cryptococcomas. These suggest the presence of trehalose-a compound that constitutes the fungal cell wall-which can point toward the fungal nature of an ambiguous focal brain lesion [53,54]. This characteristic spectral profile may facilitate differentiating a cryptococcoma from gliomas and bacterial brain abscesses. It is worth noting, however, that while the detection of a trehalose peak is a specific marker, its sensitivity is limited by the resolution of the method and depends on the fungal burden within the voxel-the signal may not reach the detection threshold in the early stages of the disease, in small lesions, or during effective antifungal therapy [85]. MRS can play an assistive role in the diagnosis and differentiation of cryptococcoma, potentially helping to avoid a brain biopsy and facilitating earlier initiation of targeted antifungal therapy.

##### Fungal Abscess

A fungal abscess is a focal infection of the brain parenchyma that leads to the formation of a necrotic center surrounded by an inflammatory capsule. Fungal abscesses mainly affect immunocompromised individuals, but they can also occur in immunocompetent patients [86]. A study by Luthra et al. comparing neuroimaging findings of abscesses of various etiologies describes, among others, 8 patients with a fungal abscess who underwent MRS [36]. The most characteristic finding turned out to be trehalose peaks between 3.6 and 3.8 ppm, which were reported in 5 out of 8 patients and did not occur in abscesses of any non-fungal etiology. Furthermore, the authors suggest that the absence of these peaks in 3 patients might have resulted from a concentration of trehalose too low to be detected by a 1.5 T MRI scanner. High lipid peaks in the range of 0.9–1.3 ppm and high lactate peaks (1.3 ppm) were frequent but non-specific findings. Amino acid peaks, which occur almost always in bacterial abscesses, were also present in 50% of cases (4/8) of fungal abscesses, which may indicate intra-abscess proteolysis. In the above study, acetate and succinate peaks—frequently observed in bacterial abscesses—were not found in any of the fungal abscesses; however, clinical cases of mucormycosis featuring acetate and succinate peaks have been reported [86]. NAA, Cho, and Cr peaks are usually absent or barely visible in fungal abscesses, and their presence may result from partial volume effects [30].

#### 3.3.5. Summary

In summary, MRS may represent a valuable adjunct to conventional MRI in the diagnosis of focal CNS infections, providing information on their metabolic profiles and facilitating their differentiation. In particular, the identification of characteristic metabolites such as trehalose, guanidinoacetate, amino acids, pyruvate, acetate, and succinate may be useful. In Table 1, we have summarized the metabolic profiles of the above-described lesions reported in the literature to facilitate their differentiation. At the same time, the use of MRS is associated with several limitations, including the often low specificity of the observed metabolic alterations, overlap between the metabolic profiles of different disease entities, and the dependence of MRS findings on lesion size and location, acquisition parameters, and the ability to accurately position the voxel. Therefore, MRS should be regarded as an adjunctive technique, and its findings should always be interpreted in conjunction with the clinical presentation and conventional MRI findings.

### 3.4. Post-Infectious Encephalopathies

#### 3.4.1. Acute Encephalopathy with Biphasic Seizures and Late Reduced Diffusion (AESD)

Interestingly, MRS may also prove clinically useful in encephalopathies caused by acute infection (most commonly HHV-6 (Human Herpesvirus 6) and HHV-7, as well as the influenza virus). A study by Takanashi et al., conducted on 16 children with excitotoxic encephalopathy (8 with AESD and 8 with MEEX–Mild Encephalopathy associated with Excitotoxicity), aimed at evaluating the prognostic value of brain metabolites, showed that a decrease in NAA in the frontal/frontoparietal white matter within the first week of AESD onset was associated with a poor neurological prognosis measured by the PCPC (Pediatric Cerebral Performance Category) scale [55]. A decrease in Cr and an increase in Lac proved to be less prognostically valuable. Furthermore, the use of MRS supports the hypothesis that AESD may result from excitotoxic damage combined with delayed or apoptotic neuronal death [56,57].

Single-voxel spectroscopy demonstrated that in the frontoparietal white matter, Glu levels rise on days 1–4 from the onset of AESD, followed by an observed increase in Gln on days 3–12 [57]. These observations may suggest a process in which an excess of Glu is released from neurons, exceeding the capacity of astrocytes to convert Glu into Gln. The increase in Glu concentration detected via MRS can occur even before the changes described in AESD become visible on standard MRI; therefore, using MRS in the early phase of AESD may facilitate the differential diagnosis of prolonged febrile seizures, and may contribute to early therapeutic intervention.

#### 3.4.2. Acute Necrotizing Encephalopathy (ANEC)

ANEC is a rare clinicoradiological syndrome most commonly triggered by common viral infections, characterized by rapid neurological deterioration and high mortality [92].

Ibrahim et al. performed MRS in 15 patients with ANEC using the single-voxel technique, placing the voxel within lesions visible on conventional MRI while avoiding areas of hemorrhage and necrosis [58]. Results in all patients in the acute phase demonstrated lipid-lactate peaks, which significantly decreased or normalized over time. Decreased NAA peaks were found in 8 out of 15 patients, and glutamate/glutamine complex peaks were observed in 4 out of 15 patients, suggesting potential excitotoxicity. The use of MRS in ANEC thus provides insight into the pathomechanism of the disease and enables monitoring of its progression, while other clinical case reports also suggest that this method may help assess the severity and prognosis of ANEC. Specifically, it was observed that the presence of the Glx peak (2.0–2.5 ppm) and a markedly elevated, broad Lip/Lac complex peak (0.8–1.5 ppm) correlate with a more severe clinical condition and high mortality [59,93]. Ibrahim et al. also proposed using MRS parameters as one of the components of a multi-parametric MRI scoring system correlating with the patient’s clinical state [58].

#### 3.4.3. Subacute Sclerosing Panencephalopathy (SSPE)

SSPE is a rare CNS disorder caused by a persistent measles virus infection. Research using MRS in this condition focuses primarily on small patient cohorts or individual case reports; however, it indicates that the application of MRS can offer several benefits in certain scenarios. The metabolic profile of CNS lesions is relatively non-specific, yet it appears to vary depending on the disease stage [60].

In the early stages of the disease, only a slight decrease in NAA peaks has been described, whereas an increase in the Cho/Cr ratio pointing to inflammation and membrane turnover was particularly notable [60]. An elevation in mI/Cr suggesting glial activation in stages II and III, followed by a subsequent drop in mI/Cr in stage IV (which may be associated with necrosis), has also been reported [61].

As the disease progresses, a decline in NAA/Cr occurs, which correlates with worsening clinical symptoms. The appearance of lipid and lactate peaks has also been documented in later stages of the disease. Consequently, MRS may allow more precise monitoring of disease progression than conventional MRI.

Furthermore, a series of 3 clinical cases reported by Cakmakci et al. indicates that MRS can detect metabolic alterations in normal-appearing white matter (an increase in mI/Cr) even prior to the onset of structural changes on MRI [62]. Seo et al. reached similar conclusions; however, they demonstrated a decrease in NAA/Cr and an increase in Cho/Cr in structurally unaffected areas of the CNS [63]. Moreover, as suggested by a case reported by Takashima et al., MRS may aid in evaluating treatment response, as they observed an increase in the previously reduced NAA/Cr ratio 6 months after initiating therapy, which correlated with clinical improvement [64].

In summary, MRS in SSPE enables the early detection of metabolic alterations before structural changes appear on conventional MRI, allows precise monitoring of disease progression, and may support the evaluation of treatment response.

### 3.5. Retroviral Infections and Opportunistic Complications

#### 3.5.1. HIV-Associated Encephalopathy/HIV-Associated Neurocognitive Disorder (HAND)

HAND comprise a spectrum of cognitive impairments ranging from mild deficits to severe dementia occurring in people living with HIV and persisting despite effective antiretroviral therapy [94]. The pathogenesis of HAND is associated with the early entry of the virus into the central nervous system (CNS). Yet HIV does not directly infect neurons; neuronal damage takes place indirectly as a result of viral proteins, chronic neuroinflammation, and dysfunction of the blood–brain barrier [95].

A meta-analysis of 27 studies involving a total of 1255 patients with HIV showed that the MRS metabolic profile in HAND is marked by elevated levels of Cho and mI, especially in the basal ganglia and in the frontal white matter. Furthermore, disease progression was linked to reduced NAA, with lower NAA levels being connected with the occurrence of cognitive impairment [96].

A study of 14 patients who had HIV-associated encephalopathy indicated that a higher mI/Cr ratio might be one of the earliest and most reliable spectroscopic indicators of HAND. However, this result was obtained from a relatively small number of patients and its diagnostic importance needs to be confirmed in larger groups [65].

MRS may be a potentially useful tool for assessing the severity of brain involvement and for monitoring the response to combination antiretroviral therapy (cART), since improvements in the metabolic parameters have been noted several months after the start of treatment. Even though cART is effective, a chronic inflammatory process may still persist in some patients, possibly causing subclinical and progressive damage to the nervous system [97].

In HAND there may also be an increase in Glx, which could be indicative of excitotoxicity and thus offer some insight into one of the mechanisms causing neuronal injury in this condition. Yet this finding is considerably less well established than the more consistently reported increases in mI and Cho and the decrease in NAA [97].

To summarize, the use of MRS in HAND may provide several possible benefits. First, the metabolic profile may reveal abnormalities before structural changes become apparent on conventional MRI. In addition, MRS may help assess the severity of CNS involvement and may be useful for monitoring the response to cART, as reductions in Cho and mI peaks have been reported following treatment initiation, potentially denoting a reduction in neuroinflammation. MRS may also provide complementary information for clinical assessment, as decreased NAA and increased mI have been associated with cognitive impairment.

#### 3.5.2. Progressive Multifocal Leukoencephalopathy (PML)

PML is a demyelinating disease of the central nervous system caused by the reactivation of the John Cunningham virus (JCV). The primary predisposing factor for the development of PML is HIV infection. Typically, MRS within MRI-visible lesions in PML demonstrates a decrease in NAA peaks along with an increase in choline, lactate, and lipid concentrations [98].

PML lesions may show varying metabolic profiles depending on their stage. Newer lesions, characterized by active demyelination, feature a higher choline peak and a less pronounced drop in NAA compared to older lesions where permanent neuronal loss has occurred [66,99].

Dominant lactate peaks can be characteristic of PML-Kozić et al. reported that they were 2 to 3 times higher than choline peaks, 4 to 6 times higher than Cr peaks, and up to 4 to 11 times higher than NAA [67]. However, these observations were based on only 3 cases, so a very marked increase in lactate cannot be considered a pathognomonic metabolic abnormality for PML.

Myo-inositol (mI) concentration, similarly to Cho, also fluctuates depending on the lesion stage. In the initial phases of PML lesion development, mI concentration rises sharply, which is associated with high glial repair activity. Interestingly, a high mI level can serve as a positive prognostic indicator associated with longer survival and disease remission. In contrast, later-stage CNS lesions are characterized by a gradual decline in the mI peak.

MRS in PML has also been applied to compare the metabolic profile of brain tissue in patients with PML-immune reconstitution inflammatory syndrome (IRIS) versus those without IRIS [68]. The chemical composition of lesions in IRIS reflected a more severe inflammatory process and tissue destruction (lower NAA/Cr ratio, higher Cho/Cr, mI/Cr, lipid/Cr, and lactate/lipid ratios).

A study by Simone et al. evaluating MRS in the differential diagnosis of CNS lesions in HIV indicates that this technique has low specificity [45]. However, they observed that PML lesions (as well as lymphomas) are characterized by a statistically significantly lower NAA/Cr ratio than lesions in toxoplasmosis and HIV encephalopathy.

Nevertheless, the described metabolic profile can overlap with other CNS diseases, and MRS should be used as a complementary tool in the differential diagnosis rather than a standalone technique.

## 4. Conclusions

Proton magnetic resonance spectroscopy (^1^H-MRS) is a valuable, non-invasive method supporting the diagnosis of inflammatory and infectious CNS disorders. It enables the detection of metabolic alterations that remain invisible on conventional magnetic resonance imaging. Metabolic disturbances demonstrated by MRS provide insight into the pathogenesis of clinical symptoms and may help resolve the clinical-radiological paradox observed in various types of encephalitis.

Our review demonstrated that the detection of specific metabolites in the spectrum can be particularly useful in the differential diagnosis of focal infectious lesions. The presence of cytosolic amino acids, acetate, and succinate supports a bacterial etiology of a brain abscess. Trehalose, on the other hand, serves as a specific marker for fungal infections (cryptococcoma, fungal abscess). The presence of guanidinoacetate and prominent lipid peaks points toward the diagnosis of tuberculoma, while varying choline concentrations help differentiate infectious/inflammatory processes from neoplastic lesions.

Studies indicate that key neurometabolite levels (e.g., decreased NAA, elevated mI, and alterations in the glutamate–glutamine complex) may correlate with the patient’s clinical state, the severity of neurological symptoms, and cognitive deficits. Serial MRS examinations may also enable monitoring of disease progression, evaluation of the reversibility of neuronal damage, and assessment of treatment response.

However, despite its significant advantages, MRS also has limitations. Existing studies indicate that metabolic profiles among different disease entities frequently overlap, thereby complicating differential diagnosis. For this reason, MRS should not be considered a standalone diagnostic tool, but rather a complementary modality to conventional MRI, other advanced neuroimaging techniques, laboratory testing, and, above all, the clinical presentation.

To fully evaluate the potential of MRS as a diagnostic adjunct, a tool for monitoring disease course and treatment response, and an indicator of patient prognosis, further large-scale, multicenter longitudinal studies in patients with neuroinfections are necessary. Furthermore, a significant challenge emerging from the literature review on MRS studies is the substantial heterogeneity of acquisition protocols and data processing methods used, as well as the widespread lack of reporting of key technical parameters (such as chemical shift displacement, shimming quality, and precise basis set modeling). Adherence to the ISMRM expert consensus methodological guidelines and technical parameter reporting standards will be crucial for future research [11,100]. Standardization of acquisition sequences (e.g., implementation of semi-LASER sequences to minimize chemical shift displacement at 3T magnetic field strength) and comprehensive reporting of methodological details will be essential for inter-center comparability and reproducibility of MRS findings in inflammatory CNS disorders.

## Figures and Tables

**Table 1:** Summary of the technical acquisition parameters of proton magnetic resonance spectroscopy and voxel locations in the studies and clinical case reports included in the review, stratified by disease etiology.

Reference	Etiology	SVS/MVS	Sequence	TR	TE	Volume [mL]	Magnetic Field [Tesla]	Number of Patients	Voxel Positioning
Sämann et al., 2003 [8]	HSE	SVS	STEAM	3000	30	7.2–8	1.5	1	right perisplenial lesion
Takanashi et al., 1997 [9]	HSE	SVS	PRESS	2000	136	8	1.5	1	right and left parietal WM
Tyrakowska-Dadełło et al., 2018 [10]	HSE	SVS	PRESS	N/A	N/A	8	1.5/3	4	lesions in both temporal lobes
Hitosugi et al., 1996 [12]	HSE	SVS	N/A	N/A	N/A	N/A	1.5	2	bilateral temporal lobes
Panagopoulos et al., 2019 [13]	HSE	SVS	N/A	N/A	N/A	N/A	N/A	1	right cerebellar hemisphere
Zawadzki et al., 2019 [14]	TBE	SVS	PRESS	2000	135	3.375	3	25 (25 HC)	bilaterally in basal ganglia, thalami and cerebellar hemispheres
Splendiani et al., 2016 [15]	anti-NMDARE	SVS	PRESS	1700	35 and 144	N/A	N/A	1	abnormal areas in the right cerebellar hemisphere (and contralateral cerebellum)
Kataoka et al., 2009 [16]	anti-NMDARE	multi-voxel	SE	1500	135	1.5	1.5	1 (5HC)	2D CSI grid at the level of basal ganglia & thalami (analyzed voxels: bilaterally basal ganglia, thalami and subcortical frontal WM)
Hudasch et al., 2026 [17]	anti-NMDARE	3D EPSI	EPSI	1550	17.6	basic: 0.107, effective: 1.5	3	20 (20 HC)	whole brain
Fortea et al., 2025 [18]	anti-NMDARE	SVS	PRESS	3000	30	dmPF: 9.0; mTL: 12.0	3	32 (36 HC)	left dmPF, left mTL
Hang et al., 2020 [19]	anti-LGI1E	SVS	N/A	N/A	N/A	N/A	N/A	1	left hippocampus
Szots et al., 2017 [20]	anti-LGI1E	SVS	PRESS	2000	30	WM: 3.375; GM: 6.0	3	9 (9 HC)	right centrum semiovale (WM), mid-occipital gray matter (GM)
Taraschenko et al., 2025 [21]	AE (anti-NMDAR, LGI1, GAD65, ab-neg)	SVS	semiLASER	5000	30	4.1–4.3	3	12 AE (11 HC)	left and right hippocampus, left prefrontal cortex
Isik et al., 2004 [22]	PLE (anti-Hu)	SVS	PRESS	2000	30 and 144	~8	1.5	1	initial: right superior temporal neocortex; follow-up: bilateral medial temporal regions (hippocampal regions)
Villanueva-Castro et al., 2025 [23]	low-grade glioma/astrocytoma/oligoastrocytoma/oligodendroglioma mimicking LE)	N/A	N/A	N/A	N/A	N/A	N/A	8	temporal lobe
Pradeep et al., 2014 [24]	RE	2D CSI	N/A	1200	33	2	1.53	2	involved parenchyma with signal changes (and contralateral normal region as reference)
Wellard et al., 2004 [25]	RE	SVS	PRESS	3000	30/35	8	3	1	temporal lobe
Sener, 2000 (abstract) [26]	RE	N/A	N/A	1500	135, 40, 20	N/A	N/A	3 (14 HC)	affected brain parenchyma
Sener, 2003 [27]	RE	hybrid CSI	PRESS	1500	40	1.76 (4 × 4 × 2 cm^3^ VOI, 16 voxels)	1.5	1 (5 HC)	affected brain parenchyma
Faria et al., 2009 [28]	RE	SVS	PRESS	1500	135	8	2	4	parietal deep white matter (bilaterally)
Sundgren et al., 1999 [29]	RE	2D CSI	SE	1500	270	1.5	1.5	1	supraventricular deep and periventricular WM (frontal and frontoparietal)
Burtscher & Holtås, 1999 [30]	pyogenic abscess	2D CSI	PRESS	1500	135, 270	N/A	1.5	3	abscess cavity (and surrounding edema)
Dev et al. 1998 [31]	pyogenic abscess	SVS	STEAM/SE	3000	20 (STEAM)/135 (SE)	4.09–8	1.5	24	abscess cavity
Kim et al., 1997 [32]	pyogenic abscess	SVS	PRESS	2000	270 (135 in selected patients)	8	1.5	7	abscess cavity
Lai et al., 2005 [33]	pyogenic abscess	SVS	PRESS	1600	135, 270	8	1.5	15	abscess cavity
Pal et al., 2010 [34]	pyogenic abscess	SVS	SE	3000	144	2.0–3.0	1.5	194	abscess cavity
Garg et al., 2004 [7]	pyogenic abscess	SVS	STEAM, SE	3000	20 (STEAM), 135 (SE)	4.09–8.00	1.5	75	abscess cavity
Himmelreich et al., 2005 [35]	pyogenic abscess	SVS	STEAM/PRESS	1500	20, 30 (STEAM)/135 (PRESS)	6–10	1.5	7	abscess cavity
Luthra et al., 2007, [36]	pyogenic abscess	SVS	SE	3000	144	2–3	1.5	91	abscess cavity
Parry et al., 2018 [37]	pyogenic abscess	3D CSI	PRESS	1690	135	N/A	1.5	2	brain parenchyma (focal lesions)
Santy et al., 2011 [38]	tuberculoma	SVS	PRESS	2000	35/144	1–8	3	4 (1 HC)	within focal lesions
Morales et al., 2015 [39]	tuberculoma	SVS	PRESS	1500	35 and 144	4–8	1.5	13 (19 HC)	lesion (mainly enhancing portions)
Parry et al., 2018 [37]	tuberculoma	3D CSI	PRESS	1690	135	N/A	1.5	72	brain parenchyma (focal lesions)
Pretell et al., 2005 [40]	tuberculoma	SVS	PRESS	1500	35/144	8	1.5	4	lesion area and contralateral side
Gupta et al., 2002 [41]	tuberculoma	SVS	STEAM/SE	3000	20/135	4–8	1.5	33	center of the lesion
Jayasundar et al., 1999 [42]	tuberculoma	SVS	STEAM	3000	135	1–8	1.5	28	within the boundaries of the lesion
Luthra et al., 2007 [36]	tubercular abscess	SVS	SE	3000	144	2–3	1.5	11	center of the lesion
Fuchs et al., 2012 [43]	tuberculous abscess/tuberculoma	SVS	N/A	N/A	135	N/A	N/A	1	center of the lesion
Barcelo et al., 2010 [44]	cerebral toxoplasmosis	SVS	N/A	N/A	35	N/A	N/A	1	lesion in the pons
Simone et al., 1998 [45]	cerebral toxoplasmosis	SVS	SE	1500	135	3.375–8	1.5	20 (22 HC)	focal lesions
Ferraz-Filho et al., 2009 [46]	cerebral toxoplasmosis	SVS	PRESS	N/A	144	3.375–8	1.5	42	inflammatory lesion
Simone et al., 1998 [45]	HIV-related encephalopathies	SVS	SE	1500	135	3.375–8	1.5	25 (22 HC)	white matter/centrum semiovale
Simone et al., 1998 [45]	CNS Lymphoma	SVS	SE	1500	135	3.375–8	1.5	7 (22 HC)	periventricular focal lesions
Ferraz-Filho et al., 2009 [46]	Glial neoplasms (Grade III-IV)	SVS	PRESS	N/A	144	3.375–8	1.5	39	lesion
Batta et al., 2020 [47]	neurocysticercosis	SVS	N/A	N/A	N/A	N/A	N/A	24	ring-enhancing lesions
Chawla et al., 2022 [48]	neurocysticercosis	SVS	PRESS	2000	30	1	3	1	right temporal lobe lesion
Kumar et al., 2023 [49]	neurocysticercosis	SVS	N/A	N/A	N/A	N/A	N/A	3	parenchymal focal lesions
Parry et al., 2018 [37]	neurocysticercosis,	3D CSI	PRESS	1690	135	N/A	1.5	6	brain parenchyma (focal lesions)
Pretell et al., 2005 [40]	neurocysticercosis	SVS	PRESS	1500	35/144	8	1.5	6	lesion area and contralateral side
Jayasundar et al., 1999 [42]	tuberculoma	SVS	STEAM	3000	135	1–8	1.5	10	within the boundaries of the lesion
Pandit et al., 2001 [50]	neurocysticercosis	SVS	PRESS	1500	35 and 144	~8	1.5	1	right cerebellar hemisphere (cystic lesion)
Batista & Gasparetto, 2012 [51]	cryptococcoma	SVS	N/A	N/A	30	N/A	N/A	1	right frontoparietal mass lesion
Uppar et al., 2024 [52]	cryptococcoma	SVS	N/A	N/A	N/A	N/A	N/A	1	lesion in right frontal lobe
Duarte et al., 2017 [53]	cryptococcoma	SVS	N/A	N/A	N/A	N/A	N/A	1	brain granuloma
Vanherp et al., 2021 [54]	cryptococcoma	SVS	STEAM	1500	30	8	1.5	2	cerebellum/parietal cortex
Luthra et al., 2007 [36]	fungal abscess	SVS	SE	3000	144	2–3	1.5	8	abscess cavity
Takanashi et al., 2020 [55]	AESD	SVS	PRESS	5000	30	4.5	3	8	fronto-parietal white matter
Hirai et al., 2017 [56]	unclassified mild encephalopathy	SVS	PRESS	5000	30	4.5	3	3	frontal white matter
Takanashi et al., 2015 [57]	AESD	SVS	PRESS	5000	30	4.5	1.5/3	5	frontal white matter
Ibrahim et al., 2020 [58]	ANEC	SVS	N/A	N/A	115	N/A	1.5	15	thalamus, cerebellar WM, basal ganglia
Goo et al., 2003 [59]	ANEC	SVS	STEAM	3000	30	7	1.5	2	bilateral thalami
Alkan et al., 2003 [60]	SSPE	SVS/CSI	PRESS	1500 (MVS)/2000 (SVS)	136 (MVS)/31 (SVS)	8	1.5	6 (10 HC)	frontal subcortical WM and parieto-occipital WM
Aydin et al., 2006 [61]	SSPE	SVS	STEAM	3500	48	N/A	N/A	20 (20 HC)	left frontal periventricular white matter
Cakmakci et al., 2004 [62]	SSPE	SVS	PRESS	2000	31 and 136	8	1.5	3	parieto-occipital, frontal, and occipital WM
Seo et al., 2010 [63]	SSPE	SVS	PRESS	2000	31	8	1.5	1	left occipital white matter
Takashima et al., 2003 [64]	SSPE	SVS	STEAM	1500	20	8	1.5	1 (5 HC)	6 VOIs: right/left frontal WM, right/left occipital WM, right/left basal ganglia
Arneth et al., 2013 [65]	HIV encephalopathy	SVS	STEAM	1500	20	8	1.5	14	parietal WM, occipital GM, frontal WM
Yoon et al., 2007 [66]	PML	SVS	N/A	N/A	N/A	N/A	N/A	1	white matter lesions
Kozić et al., 2017 [67]	PML	SVS	PRESS/STEAM	2000	135 and 20	8	3	3	white matter/subcortical lesions
Gheuens et al., 2012 [68]	PML/PML-IRIS	SVS	PRESS	2000	35	8	3	42	center of the PML lesion
Simone et al., 1998 [45]	PML	SVS	SE	1500	135	3.375–8	1.5	8 (22 HC)	multifocal white matter lesions

Abbreviations used in the table: 2D CSI—Two-Dimensional Chemical Shift Imaging, 3D CSI—Three-Dimensional Chemical Shift Imaging, 3D EPSI/EPSI—Three-Dimensional Echo-Planar Spectroscopic Imaging, ab-neg—Antibody-Negative, AE—Autoimmune Encephalitis, AESD—Acute Encephalopathy with Biphasic Seizures and Late Reduced Diffusion, ANEC—Acute Necrotizing Encephalopathy of Childhood, anti-Hu—Anti-Hu Antibodies, anti-LGI1E—Anti-Leucine-Rich Glioma-Inactivated 1 Encephalitis, anti-NMDARE/anti-NMDAR—Anti-N-Methyl-D-Aspartate Receptor Encephalitis, CNS—Central Nervous System, dmPF/dmPFC—Dorsomedial Prefrontal Cortex, GAD65—Glutamic Acid Decarboxylase 65, GM—Gray Matter, HC—Healthy Controls, HIV—Human Immunodeficiency Virus, HSE—Herpes Simplex Encephalitis, LE—Limbic Encephalitis, LGG—Low-Grade Glioma, mTL—Medial Temporal Lobe, MVS—Multi-Voxel Spectroscopy, N/A—Not Applicable/Not Available, PLE—Paraneoplastic Limbic Encephalitis, PML—Progressive Multifocal Leukoencephalopathy, PML-IRIS—Progressive Multifocal Leukoencephalopathy—Immune Reconstitution Inflammatory Syndrome, PRESS—Point Resolved Spectroscopy, RE—Rasmussen’s Encephalitis, SE—Spin Echo, semiLASER—Semi-Localized by Adiabatic Selective Refocusing, SSPE—Subacute Sclerosing Panencephalitis, STEAM—Stimulated Echo Acquisition Mode, SVS—Single-Voxel Spectroscopy, TBE—Tick-Borne Encephalitis, TE—Echo Time, TR—Repetition Time, VOI—Volume of Interest, WM—White Matter.

**Table 2:** The table presents the metabolic profile in magnetic resonance spectroscopy (MRS) in the differential diagnosis of CNS infectious lesions (bacterial, fungal, and parasitic), which facilitates their differentiation. Features that may prove particularly useful in differentiating individual focal lesions are highlighted in red in the table. Numerical values for metabolite ratios represent mean values (± standard deviation) or ranges (minimum–maximum) as reported in the cited studies.

Metabolic Marker	Brain Abscess (Bacterial)	Tuberculoma	Cerebral Toxoplasmosis	Neurocysticercosis	Cryptococcosis	Fungal Abscess
NAA (2.0 ppm)	↓↓/absence	↓↓	↓↓	↓	↓	↓↓/absence
Cr (3.0 ppm)	↓↓/absence	↓↓	↓↓	↓	↓	↓↓/absence
Cho (3.2 ppm)	↓↓/absence	may be ↑	↓/↔/↑	↓/↔/↑	↑	↓↓/absence
**Cho/Cr**	NAA, Cr, and Cho) are absent or severely degraded within the necrotic core of bacterial abscesses, rendering metabolite ratios non-measurable and non-diagnostic	↑ * 1.3 (0.8–2.4) (Morales et al., 2015) [39] **1.36** ± **0.41** (Parry et al., 2018) [37] **2.13**; compared to normal brain tissue, the ratio was **0.68** (Pretell et al., 2005) [40]	↑ **1.61** ± **0.76** (Simone et al., 1998) [45]	**0.88**, compared to normal brain tissue, the ratio was **0.73** (Pretell et al., 2005) [40] **0.87** (Kumar et al., 2023) [49]	may be ↑	N/A
**NAA/Cho**		↓ **0.64** ± **0.23** (Parry et al., 2018) [37] **0.81** ± **0.62** (Jayasundar et al., 1999) [42]	↓ **1.09** ± **0.56** HC: 2.09 ± 0.39 (Simone et al., 1998) [45]	**0.30** ± **0.61** (Jayasundar et al., 1999) [42]	N/A	N/A
**NAA/Cr**		↓ **1.2 (0.8–1.5)** (Morales et al., 2015) [39] **0.83** ± **0.30** (Parry et al., 2018) [37] **1.74**, compared to normal brain tissue, the ratio was **0.58** (Pretell et al., 2005) [40] **1.47** ± **0.95** (Jayasundar et al., 1999) [42]	↓ **1.47** ± **0.36** HC: 2.25 ± 0.51 (Simone et al., 1998) [45]	↔/↓ **1.69,** compared to normal brain tissue, the ratio was **0.74**) (Pretell et al., 2005) [40] **0.12** ± **0.27** (Jayasundar et al., 1999) [42]	N/A	N/A
**Cho/NAA**		↑ **1.56** (Pretell et al., 2005) [40]	↑ **0.92** (Simone et al., 1998) [45]	↑	may be ↑	N/A
**mI** (3.5–3.6 ppm)	absence	↓/↔	may be ↓	N/A	N/A	N/A
**Lipids** (0.9, 1.3, 2.0, 2.8 ppm)	may be present	**Present in 75–86% tuberculomas** **(0.9 and/or 1.3 ppm)**	↑	usually absent	May be ↑	↑
**Lac** (1.3 ppm)	↑↑ always present	may be present (42% of tuberculomas)	↑	may be present	May be ↑	usually ↑
**Amino acids** (alanine at 1.47 ppm, leucine at 0.95–1.05 ppm, and valine at 0.97–1.03 ppm)	↑ **almost always** **(80–100%)**	absence	absence	may be present alanine (alanine, BCAA (~0.9 ppm)	not characteristic	May be present
**Succinate** (2.4 ppm)/**acetate** (1.9 ppm)	**present in 35% and 85% of brain abscesses, respectively** **(acetate > succinate)**	absence	absence	**Present** **(one or both, optionally with alanine and pyruvate)** (succinate > acetate)	not characteristic	Rarely may be present
Additional informations	-	**A characteristic guanidinoacetate peak may be present at 3.8 ppm**			**trehalose peak (3.6–3.8 ppm) may be present**	
References	[7,31,32,34,35]	[36,37,38,39,79,80,81]	[44,45,46,82]	[47,48,49,83]	[51,52,53,54,84,85]	[30,36,86]

* The significant difference between the results may stem from the fact that Pretell et al. used a fixed voxel volume (8 mL), which might have also included areas of brain parenchyma affected by the inflammatory process, whereas Morales et al. adjusted the voxel size to the dimensions of the tuberculoma; additionally, the study group in Pretell et al. was significantly smaller (*n* = 4) compared to Morales et al. (*n* = 13) and Parry et al. (*n* = 72). Abbreviations and symbols used in the table: NAA (N-acetylaspartate), Cr (creatine/phosphocreatine), Cho (choline), Cho/Cr (choline-to-creatine ratio), mI (myo-inositol), Lipids, Lac (lactate), BCAA (branched-chain amino acids), ppm (parts per million-chemical shift unit), N/A (not applicable), ↑—increased, ↓—decreased, ↑↑—severely increased, ↓↓—severely decreased, ↔—remained unchanged.

## Data Availability

No new data were created or analyzed in this study. Data sharing is not applicable to this article.
